# Stochastic switching of TiO_2_-based memristive devices with identical initial memory states

**DOI:** 10.1186/1556-276X-9-293

**Published:** 2014-06-10

**Authors:** Qingjiang Li, Ali Khiat, Iulia Salaoru, Hui Xu, Themistoklis Prodromakis

**Affiliations:** 1 College of Electronics Science and Engineering, National University of Defense Technology, Changsha 410073, China; 2 Southampton Nanofabrication Centre, Nano Group, Department of Electronics and Computer Science, University of Southampton, Southampton SO17 1BJ, UK

**Keywords:** Resistive switching, Initial state, Filamentary distribution

## Abstract

In this work, we show that identical TiO_2_-based memristive devices that possess the same initial resistive states are only phenomenologically similar as their internal structures may vary significantly, which could render quite dissimilar switching dynamics. We experimentally demonstrated that the resistive switching of practical devices with similar initial states could occur at different programming stimuli cycles. We argue that similar memory states can be transcribed via numerous distinct active core states through the dissimilar reduced TiO_2-*x*_ filamentary distributions. Our hypothesis was finally verified via simulated results of the memory state evolution, by taking into account dissimilar initial filamentary distribution.

## Background

Among numerous candidates for the non-volatile memories, resistive random access memory (ReRAM) is highly considered for its advantageous attributes [1-3]. Nonetheless, the operation mechanism of ReRAM devices remains a bone of contention [4,5] with the formation and rupture of conducting filaments being ascertained as the functional switching mechanism [6]. Understanding their switching dynamics is thus of critical importance for the future implementation of ReRAM. Surprisingly, there exist numerous studies that highlight the stochastic switching in ReRAM [7-10]. In [8], the experimental results show that both the distributions of *I*_RESET_ and *V*_RESET_ are strongly influenced by the distribution of initial resistance. In addition, Shibuya et al. [11] have demonstrated the impact of pristine defect distribution on current-voltage (*I*-*V*) characteristics of Sr_2_TiO_4_ thin films, demonstrating that the density of distinct initial defects would result in two opposite *I*-*V* switching polarities.

One might expect that identical ReRAM devices that possess the same initial effective resistance would attain the same resistive state evolution when provided the same programming stimulus. Nevertheless, this does not always hold for practical devices. In practical devices, randomly distributed local imperfections could act as conductive percolation branches within the devices' active cores. Such conditions employ the devices with a high probabilistic nature, which could provide very dissimilar switching characteristics. In this study, we experimentally demonstrated stochastic resistive switching in TiO_2_-based ReRAM devices that possess identical initial resistive states. We further explore the origin of this phenomenon by employing a random circuit breaker (RCB) network model [9,12]. We show that ReRAM devices that have the same initial resistance would attain distinct initial filament distributions, which would finally result in very dissimilar resistive switching dynamics even when programmed with the same pulse schemes.

## Methods

### Fabrication of TiO_2_-based active cells

In this study, we employed the following fabrication process flow. Firstly, 200-nm-thick SiO_2_ was thermally grown on a 4-in. silicon wafer. Then, e-gun evaporation was employed to deposit 5-nm Ti and 30-nm Pt that serve as adhesion and bottom electrode (BE) layers, respectively. The stoichiometric TiO_2_-based layer with a total thickness of 31 nm was then deposited by RF magnetron sputtering at 300 W and with an Argon gas flow of 30 sccm. Subsequently, a 30-nm-thick Pt top electrode (TE) film was deposited by e-gun evaporation. Optical lithography and lift-off process were adopted to define the patterns of each layer. The design allows having Pt/TiO_2_/Pt ReRAM structures in crossbars and stand-alone configurations. In this manuscript, the tested devices possess a stand-alone crossbar configuration with an active area of 5 × 5 μm^2^.

### Electrical measurements

Electrical measurements for active cells on wafer were performed utilizing a low-noise Keithley 4200 semiconductor characteristic system (Keithley Instruments Inc., Cleveland, OH, USA) combined with a semi-automatic probe station (Wentworth AVT 702, Wentworth Laboratories, Inc., Brookfield, CT, USA). During measurements, the programming voltage bias was applied to the TE, while keeping the BE grounded. The unipolar *I*-*V* characteristics were firstly attained via sweeping potentials from 0 to 5 V in steps of 0.1 V and then back to 0 V. To capture the switching dynamics of devices, a series of programming (5 V) pulses were applied across the active cells followed by a 0.5-V pulse to read the resistance values. The width durations for programming and evaluating pulses were set to 10 and 1 μs, respectively. In addition, the compliance current was set to 1 mA to avoid any hard breakdown of the devices.

### Modeling and simulations

The active core of ReRAM was modeled with a two-dimensional 20 × 20 random circuit breaker (RCB) network. Within the network, the stoichiometric TiO_2_ was represented by high-valued resistors (8 MΩ), while the conductive TiO_2-*x*_ was modeled by low-valued resistors (1 KΩ). To capture the simulated evolution of resistive state, a constant 0.5 V was applied to render the formation and rupture of filaments within the network. The RCB network was established on Matlab R2012b and then created in a PSPICE circuit. In each simulation cycle, Candence PSPICE 16.5 was called from Matlab to simulate the network with results being collected and analyzed utilizing Matlab.

## Results and discussion

**Figure 1:**
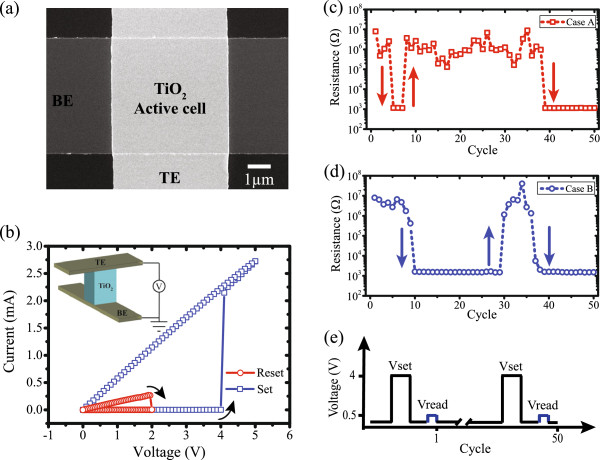
**Measured features of TiO**_**2**_**-based ReRAM devices. (a)** SEM image of a crossbar-type prototype based on TiO_2_ cell with an active area of 5 × 5 μm^2^. **(b)** Measured *I*-*V* characteristics showing a typical unipolar switching signature. Inset: schematic view of the measured cell. **(c, d)** Resistance evolution results of two practical devices with identical initial resistive states at room temperature. **(e)** Pulse-induced programming and evaluating scheme, where *V*_set_ and *V*_read_ represent resistance programming and evaluating pulses, respectively.

Figure 1a depicts a scanning electron microscope (SEM) image of a metal-insulator-metal (MIM) prototype in crossbar architecture with a 5 × 5 μm^2^ TiO_2_-based active core sandwiched between a Pt top (TE) and bottom electrode (BE), as illustrated in the inset of Figure 1b.

Initially, to investigate the switching properties, we employed quasi-static sweeping potentials with *I*-*V* curves being shown in Figure 1b, which is a typical unipolar switching signature. A reset potential of +2 V switched the device from low resistive state (LRS) to high resistive state (HRS), while an opposite switching trend occurred at +4 V in the following programming cycle. In this study, the stochastic resistive switching phenomenon was investigated only under unipolar switching mode via a voltage pulsing and evaluation scheme illustrated in Figure 1e. For each cycle, a 4-V pulse with 10-μs width was applied to switch the devices; the resistive state value was then evaluated by a pulse of 0.5 V and 1 μs, which does not disturb the intrinsic resistive state. Intriguingly, though biased with the same pulse-induced scheme, distinct switching trends were observed for two identical TiO_2_-based ReRAM cells with similar initial resistance (both *R*_INI_ = 8 MΩ), as demonstrated in Figure 1c,d. Specifically, device A required less programming cycles in the first two switching events to toggle between HRS and LRS; it switched at the 5th cycle and switched back at the 8th cycle, while for device B, similar switching events occurred at the 10th and the 30th cycles, respectively. In contrast, device B switched relatively faster (37th cycle) than device A (39th cycle) in the case of the third switching event. In this manuscript, all tested devices were electrically characterized without employing any post-fabrication electroforming step, which enhances the device interoperability with low-voltage CMOS technologies.

The stochastic switching in this research was investigated only under unipolar switching mode. Thus, the active core of our prototypes only undergoes a reduction from TiO_2_ to TiO_2-*x*_, after employing a number of pulses that induce a cumulative thermally driven mechanism [12,13]. In contrast to the bipolar switching model where resistive switching is attained via displacement of ionic species (a well-controlled stable process), unipolar switching is mainly ascribed to a thermally driven reduction of TiO_2_, which may cause inconsistent switching [14]. As a result, the activation energy supplied by a single set pulse is not sufficient to generate formation and rupture of continuous filaments. Resistive switching events are thus not available at each programming pulse, as demonstrated in Figure 1c,d. The aim of pulse-induced measurement in this manuscript is to supply well-controlled identical activation energies to the thermally driven filamentary formation and rupture procedure [14], which makes it possible to only investigate the influence of initial filament distribution on stochastic switching.

**Figure 2:**
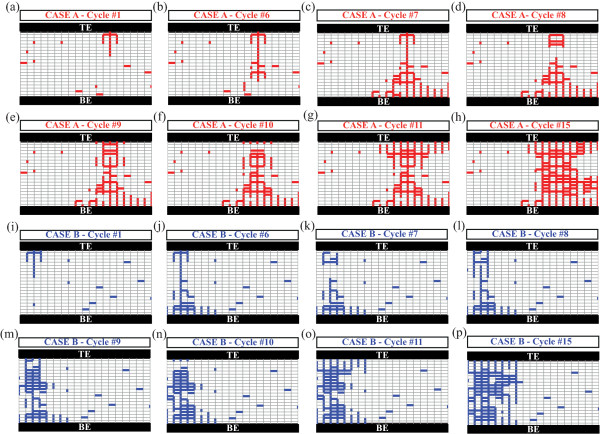
**State evolutions of two cases with identical initial resistive states.** A constant bias of 0.5 V was applied for each simulation cycle throughout **(a-h)** for case A and **(i-p)** for case B, respectively.

Here we present the relation between the resistive state and filament distribution by investigating two particular cases based on the RCB network model [12]. As illustrated in Figure 2, the thin gray grids represent stoichiometric TiO_2_ via high-value resistors (8 MΩ), while the thick red branches represent reduced TiO_2-*x*_ as conductive filaments (1 KΩ). Two special cases (A and B, as depicted in Figure 2a,i) were established with identical initial resistance (6.52 MΩ), yet for the same programming scheme, dissimilar filament distributions (defect density and path) were attained. It should be noted that devices with identical initial resistive state could attain infinite plausible cases of dissimilar filament distributions, though only two particular cases were investigated here. Clearly, the relation between the initial resistive state and the distribution of the filaments cannot be established.

**Figure 3:**
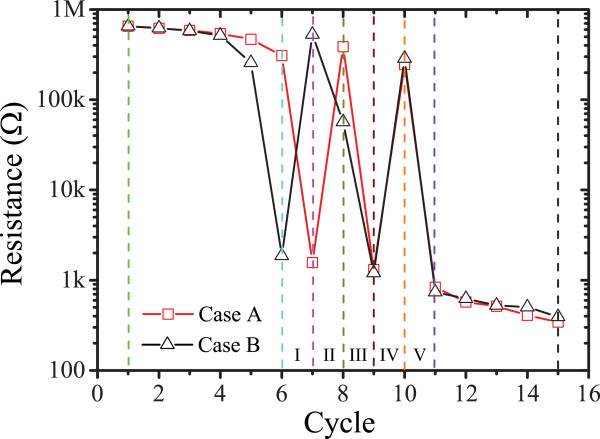
**Detailed resistance evolutions of two simulated cases.** The colored dashed lines highlight the effective resistance of all the resistive switching cycles.

In the case of our particular TiO_2_-based ReRAM cells, external stimulus would drive and distribute the defects, namely oxygen vacancies and/or titanium interstitials, randomly into the devices' active cores, which would contribute to the formation of percolation branches. Therefore, practical ReRAM devices with identical initial resistance may attain distinct filament distribution. We thus argue that such devices might attain distinct switching dynamics even when biased with the same switching protocols.Initially, case A and case B were established with dissimilar filamentary distributions, but both possess the same effective resistance of 6.52 MΩ. The devices were biased with the external stimuli that would form and rupture conductive branches within devices' active cores which would introduce the evolution of the resistive states. Key resistive switching cycles were selected, and their corresponding resistive states are shown in Figure 2. The evolution of both networks was monitored through their corresponding transient responses to the networks' effective resistance, and to allow a better visibility of the switching trends, the effective resistance of each step is depicted in Figure 3.

Clearly, case A switched from HRS to LRS at the seventh cycle, while a similar resistive switching event occurred on the sixth cycle for case B, indicating that case B required less stimulus cycles to attain the formation of continuous conductive filaments. Following these events, most of the energy provided in the consecutive cycles is dissipated through the thin formed filaments that in turn cause their fusing via Joule heating [13]. This event occurred during the eighth and seventh cycles for the cases A and B, respectively, when there is a sharp resistance increase; their corresponding network topologies are shown in Figure 2d,k. From then on, both cases A and B experienced similar state evolution (switching events III, IV, and V), but unlike the first two switching events (I and II), cases A and B require the same activation energy for forming and rupturing the percolation filaments in the following switching events. Detailed resistive switching events occurred at cycles 9, 10, and 11 with corresponding filament distribution illustrated in Figure 2e,f,g and Figure 2m,n,o for cases A and B, respectively. Finally, both cases A and B remain at similar LRS which is consistent with the measured results, since the conductive TiO_2-*x*_ is dominant in active cores after a number of programming cycles and the devices are approaching their endurance limits. It is worthy to point out that for specific switching events, the set or reset transition could be closely related to its previous state [8,9]. Nonetheless, as illustrated in Figure 2, the corresponding defect distributions in cycle 15 (Figure 2h,p) are very dissimilar for the two studied cases (A and B), yet they exhibit identical LRS. Clearly, if a reverse biasing polarity was used to reset the device in both cases to HRS, similar stochastic switching trends to the ones depicted in Figure 3 will most probably be exhibited. It should be noted that the above switching dynamics may only hold for the assumed current percolation circuit model. In practical ReRAM devices, multiple filaments may be formed and ruptured concurrently, which result in a much more complex behavior where antagonistic bipolar and unipolar switching occurs stochastically. It is also worthy pointing out that the stochastic switching characteristics could be correlated to the cell size [7] and ambient temperature [12,13]. It is anticipated that scaling the devices in submicron dimensions would in principle restrict the defect density and distribution variances, while at the same time, heat accumulation due to ambient temperature could accelerate the switching process.

## Conclusion

In conclusion, we have experimentally demonstrated that practical TiO_2_-based ReRAM devices with identical initial resistive states could exhibit very dissimilar switching dynamics. Although identical devices could possess phenomenologically similar initial states, we have demonstrated experimentally that their resistive switching occurs at different programming cycles. Finally, our hypothesis was confirmed by simulating the evolution of two distinct scenarios of a device possessing the same initial state but having dissimilar filamentary distribution. We thus argue that all ReRAM that exhibit a filamentary type of mechanism could possess stochastic switching characteristics, though our study only exploits TiO_2_-based devices. Considering the further ReRAM development, this impact of defect distribution should be carefully considered in device engineering as it could significantly affect the fabrication reproducibility and the accurate control of the devices' states, necessitating fault-tolerant design paradigms. It is possible to suppress the defects' broad distribution in TiO_2_-based pristine devices via annealing [15], although this extra processing step is not always preferable.

## Competing interests

The authors declare that they have no competing interests.

## Authors' contributions

LQ, AK, IS, XH, and TP conceived the experiments. AK and TP fabricated the samples. LQ performed the electrical characterization of the samples and simulations. All authors contributed in the analysis of the results and in the writing of the manuscript. All authors read and approved the final manuscript.
